# Evolution of Leaf Chlorophylls, Carotenoids and Phenolic Compounds during Vegetation of Some Croatian Indigenous Red and White Grape Cultivars

**DOI:** 10.3390/plants13070971

**Published:** 2024-03-27

**Authors:** Marina Anić, Jasminka Karoglan Kontić, Nera Rendulić, Mate Čarija, Mirela Osrečak, Marko Karoglan, Željko Andabaka

**Affiliations:** 1 Division of Horticulture and Landscape Architecture, Department of Viticulture and Enology, Faculty of Agriculture, University of Zagreb, Svetošimunska Cesta 25, 10000 Zagreb, Croatia; manic@agr.hr (M.A.); jkkontic@agr.hr (J.K.K.); huzanic.nera@gmail.hr (N.R.); mosrecak@agr.hr (M.O.); zandabaka@agr.hr (Ž.A.); 2 Centre of Excellence for Biodiversity and Molecular Plant Breeding, Faculty of Agriculture, University of Zagreb, 10000 Zagreb, Croatia; 3 Institute for Adriatic Crops and Karst Reclamation, Put Duilova 11, 21000 Split, Croatia; mate.carija@krs.hr

**Keywords:** grapevine, leaf carotenoids, leaf phenolic compounds, leaf chlorophylls

## Abstract

During the ripening process of grapes, the grapevine leaves are the most active green organs that are important for photosynthesis, which is closely linked to the development and metabolism of the plant. The detection of plant pigments and phenolic compounds in grapevine leaves can be a good indicator of the ageing process, vine vigor and the plant’s ability to respond to fungal attack. In a one-year study, the development of leaf chlorophylls, carotenoids and phenolic compounds during the ripening of six indigenous Croatian grape cultivars and the international cultivars Merlot and Chardonnay was investigated. The chlorophyll a/b ratio and total chlorophyll and total carotenoid concentrations were also investigated. PCA was used to highlight relevant information from the data with the aim of distinguishing individual compounds based on the cultivar and phenological stage. The leaf total hydroxycinnamic acid and flavan-3-ol concentrations decreased slowly during grape development, with the highest concentration immediately after flowering and the lowest during grape ripening. The concentrations of β-carotene, lutein and xanthophylls tended to decrease during bunch closure or veraison, while the concentration of chlorophylls a and b peaked during veraison and then decreased during grape ripening. This research will provide an opportunity to select cultivars with the physiological adaptation to synthesize secondary metabolites that are important for managing stress conditions.

## 1. Introduction

Grapevine leaves contain numerous secondary plant metabolites, in particular, phenolic compounds, chlorophylls and carotenoids. The detection of these secondary metabolites in grapevine leaves is of great interest as they are a good indicator of vine vigor, senescence, stress due to water scarcity or the occurrence of fungal diseases and are often used in precision viticulture [1].

Chlorophylls are green pigments found in all photosynthetic plants and are the basis for photosynthesis, as they convert solar energy into chemical energy that is used to build essential carbohydrate molecules (glucose), which serve as a food source for the entire plant. Chlorophylls are found in the chloroplasts, in almost all green parts of a plant, the leaves and stems [2]. Chlorophyll accumulation in grapevines varies according to the phenological stage: it increases rapidly with leaf growth until flowering, then stabilizes and may gradually decrease until the end of grape ripening [3]. Environmental conditions can affect the physiological processes of the plant, e.g., chlorophyll accumulation, gas exchange and photosynthesis in grapevine leaves [4,5], with different cultivars growing under the same or similar environmental conditions having different chlorophyll levels [6].

Carotenoids are lipid-soluble plant pigments that are responsible for various biological functions. They are synthesized in the chloroplasts and chromoplasts of fruits and vegetables, where they play an active role in protecting plants from photodamage [7]. When too much oxygen is produced during photosynthesis in response to increased light intensity, it becomes toxic through the formation of free radicals, so carotenoids, particularly lutein and β-carotene, act as singlet oxygen scavengers [8]. They are also involved in dissipating excess light energy and converting it to heat by converting violaxanthin to zeaxanthin via antheraxanthin in the so-called xanthophyll cycle, a process that is controlled by increased light intensity. When the light intensity decreases, zeaxanthin is converted back into violaxanthin [8]. Carotenoids play an important role as plant pigments and give flowers and fruits a yellow to red color [9].

Polyphenolic compounds, together with phenolic acids, are a large group of plant secondary metabolites that can be categorized according to their structure. Grapevine leaves contain different types of phenolic compounds such as phenolic acids (both hydroxybenzoic and hydroxycinnamic), flavonols, flavan-3-ols, anthocyanins and resveratrol, which play important roles in growth, reproduction and defense reactions in plants [5,10]. Grapevine leaves are the first to respond to fungal infections by *Botrytis cinerea*, *Erysiphe necator* and *Plasmopara viticola*, and the response of plants to these infections is highly dependent on the concentration and composition of phenolic compounds in the leaves [10]. The synthesis of polyphenols in grapevine leaves can also be triggered by other stress conditions, such as drought [11] and increased UV radiation, as they act as UV filters and protect the plant cells (especially the chloroplasts) from the harmful effects of UV rays [5,12,13].

The degradation of leaf pigments and polyphenols during senescence impairs plant development and metabolism and reduces the process of photosynthesis [4]. The topographic characteristics of the vineyard as well as the orientation of the vine rows, the vine vigor and the different viticultural practices can lead to great variability in microclimatic conditions and influence the concentration of carotenoids, chlorophylls and polyphenols in the leaves, as they directly affect the temperature, humidity and other environmental factors that also influence the growth of the vines [1,6,14]. Solar radiation stimulates the photosynthetic activity of plants and consequently influences the biosynthesis of these compounds [15]. Ultraviolet (UV) radiation, including UV-A (315–400 nm) and UV-B (280–315 nm), represents a very small part of the solar spectrum but has a very large biological effect. The amount of UV radiation changes depending on altitude, latitude, season, time of day and clouds [16]. However, due to the thinning of the ozone layer, UV radiation is increasing and becoming more harmful, leading to physiological changes in plants. Grapevine is well adapted to UV radiation and is used as a model plant for studying the effects of UV radiation [17]. The biosynthesis of some secondary metabolites, especially polyphenols such as flavonols, is stimulated by UV radiation [14,17,18]. Carotenoids are an important part of the UV response machinery of the vine, as they act as pigments for light harvesting [16] and an increase in UV radiation leads to increased levels of photoprotective carotenoids [19,20]. The leaf chlorophyll content is affected by heat stress, as grapevine physiological processes are temperature dependent [5].

Although mainly used as an organic fertilizer and incorporated into the soil, grapevine leaves contain many bioactive compounds that have a positive effect on human health, including carotenoids, chlorophylls and phenolic compounds, all of which have antioxidant, antimicrobial and anticancer properties. They are also an important source of vitamins A, C, E and K in the human diet, which are important for the eyes and immune system and are often used as food colorants as well as in the pharmaceutical industry [2,7,21].

The physiological responses related to the leaf secondary metabolites phenolic compounds, chlorophylls and carotenoids of Croatian indigenous red and white *Vitis vinifera* L. cultivars and the international cultivars Chardonnay and Merlot were recorded at the five stages of grape development—after flowering, during berry setting, bunch closure, veraison and grape ripening—during one growing season (2017) to investigate to what extent the cultivar influences the concentration of secondary leaf metabolites depending on the phenological stage. This study provides new insights into how indigenous cultivars can be highly responsive and physiologically efficient, with the ability to increase the concentration of compounds with protective and regulatory effects.

## 2. Results

### 2.1. Climatic Conditions and Phenological Stages

In 2017, a range of climate parameters were assessed from May to September (Table 1). The warmest and driest period and the highest sum of UV radiation were measured in August (24.21 °C, 48.1 mm and 1673.75 W/m^2^), which is considered slightly higher air temperatures for the biological processes of the plants and the ripening of the grapes. Under the climatic conditions of 2017, full bloom occurred between 30 May and 6 June, depending on the cultivar (Table 2). The early-ripening cultivars started veraison on 7–9 August, while the mid- to late-ripening cultivars started veraison 5–7 days later (14 August). Due to the different ripening dynamics of the cultivars, the last phenological stage was mid-ripening, which took place between 27 August and 5 September.

### 2.2. Concentration of Phenolic Compounds in Grapevine Leaves

**Figure 1:**
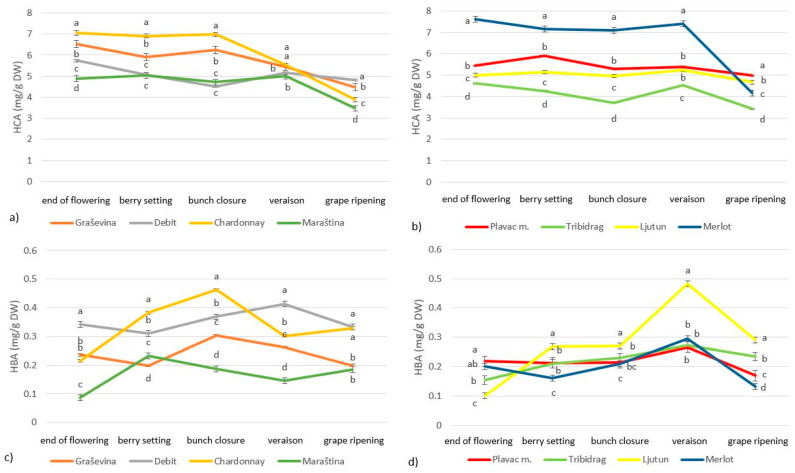
Variation in leaf total HCA (**a**,**b**) and HBA (**c**,**d**) concentration during phenological stages of *Vitis vinifera* L. white (**a**,**c**) and red (**b**,**d**) cultivars; letters indicate statistically significant difference between variables within sampling dates, with LSD test (*p* < 0.05); mean (*n* = 3) ± SE.

**Figure 2:**
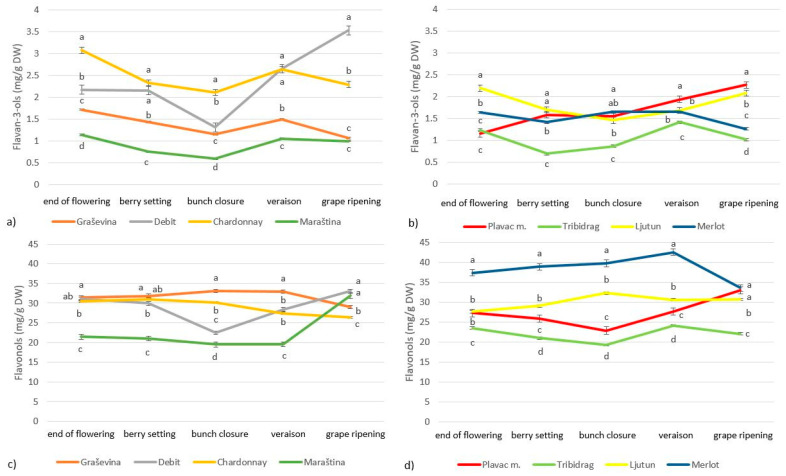
Variation in leaf total flavan-3-ol (**a**,**b**) and flavonol (**c**,**d**) concentration during phenological stages of *Vitis vinifera* L. white (**a**,**c**) and red (**b**,**d**) cultivars; letters indicate statistically significant difference between variables within sampling dates, with LSD test (*p* < 0.05); mean (*n* = 3) ± SE.

In this study, we measured the concentrations of phenolic compounds, carotenoids and chlorophylls in grapevine leaves during five stages of grape development in six indigenous Croatian grape cultivars and the international cultivars Merlot and Chardonnay. Among the phenolic compounds, total flavonols, flavan-3-ols, hydroxycinnamic acids (HCA) and hydroxybenzoic acids (HBA) were analyzed. The most abundant group of phenolic compounds were flavonols, ranging from 19 to 42 mg/g of DW, followed by HCA (3.2 to 7.6 mg/g of DW), flavan-3-ols (0.7 to 3.5 mg/g of DW) and HBA (0.1 to 0.5 mg/g of DW) (Figure 1 and Figure 2). The white cultivars had higher concentrations of total HBA and flavan-3-ols, while the concentrations of total HCA and flavonols were similar in the red and white cultivars.

The total HCA concentration decreased during grape development, with the highest concentration immediately after flowering and during berry setting and the lowest concentration during grape ripening (Figure 1). The highest total HCA concentration during grape ripening was recorded in the indigenous Croatian cultivars Debit and Plavac mali. Total HBA concentration varied significantly during grape development, with the highest concentration observed during bunch closure (white cultivars) and veraison (red cultivars), followed by a slow decline during grape ripening. The highest concentration of total HBA during grape ripening was observed in the white cultivars Chardonnay and Debit.

The concentration of total flavan-3-ols during grape development decreased slowly in most cultivars (Chardonnay, Graševina, Maraština, Ljutun, Merlot and Tribidrag), with the highest concentration observed immediately after flowering and the lowest concentration observed during grape ripening, apart from the indigenous Croatian cultivars Debit and Plavac mali, which had the highest concentration of total flavan-3-ols during grape ripening (Figure 2). The concentration of total flavonols showed considerable variation during grape development, with the concentration decreasing in some cultivars and increasing in others. The highest concentration of total flavonols during grape ripening was found in the white cultivars Debit and Maraština and red cultivars Merlot and Ljutun.

### 2.3. Concentration of Chlorophylls in Grapevine Leaves

**Figure 3:**
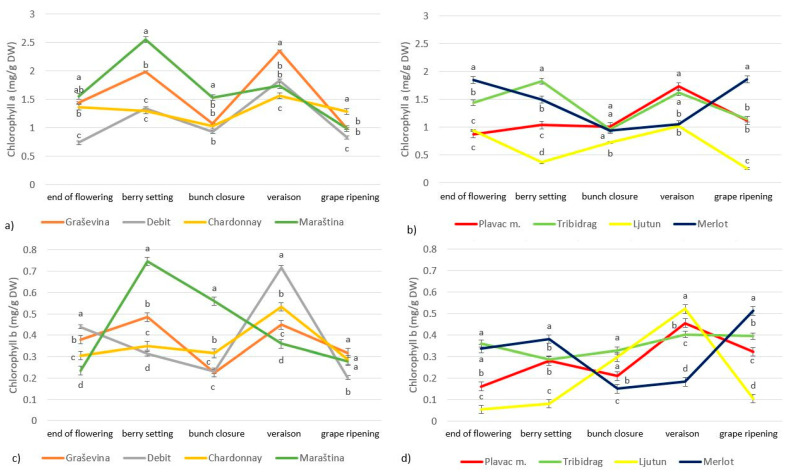
Variation in leaf chlorophyll a (**a**,**b**) and chlorophyll b (**c**,**d**) concentration during phenological stages of *Vitis vinifera* L. white (**a**,**c**) and red (**b**,**d**) cultivars; letters indicate statistically significant difference between variables within sampling dates, with LSD test (*p* < 0.05); mean (*n* = 3) ± SE.

The chlorophyll a concentration in the leaves of the red cultivars Merlot, Plavac mali, Tribidrag and Ljutun and the white cultivars Chardonnay, Maraština, Graševina and Debit ranged from 0.25 to 2.50 mg/g of DW, while the chlorophyll b concentration ranged from 0.05 to 0.75 mg/g of DW (Figure 3). The highest concentration of chlorophyll a and b in most cultivars was observed during veraison, followed by a decrease during grape ripening. Only Maraština and Tribidrag had the highest concentration of chlorophyll a during berry setting but also showed a decrease during grape ripening, and Merlot had the highest concentration during grape ripening.

### 2.4. Concentration of Carotenoids in Grapevine Leaves

**Figure 4:**
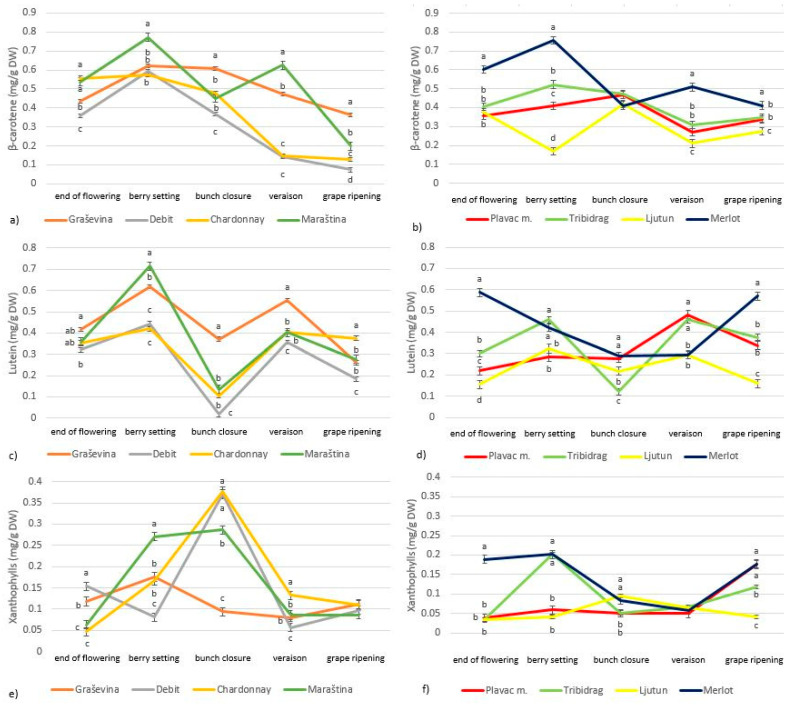
Variation in leaf β-carotene (**a**,**b**), lutein (**c**,**d**) and xanthophyll (**e**,**f**) concentration during phenological stages of *Vitis vinifera* L. white (**a**,**c**,**e**) and red (**b**,**d**,**f**) cultivars; letters indicate statistically significant difference between variables within sampling dates, with LSD test (*p* < 0.05); mean (*n* = 3) ± SE.

Among the carotenoids, the concentrations of β-carotene, xanthophylls and lutein were analyzed (Figure 4). The most abundant carotenoids were β-carotene and lutein, both in the range of 0.05 to 0.8 mg/g of DW, while xanthophylls were in the range of 0.05 to 0.4 mg/g of DW. All white cultivars and most red cultivars had the highest levels of β-carotene, lutein and xanthophylls during berry setting and bunch closure, followed by a decrease during grape ripening, apart from the red cultivar Plavac mali, which had the highest levels of lutein during veraison and xanthophylls during grape ripening. The highest concentrations of β-carotene, xanthophylls and lutein during grape ripening were found in the cultivar Merlot.

### 2.5. Leaf Chlorophyll a/b Ratio and Total Chlorophyll and Carotenoid Concentrations in Grapevine Leaves

The changes in the leaf chlorophyll a/b ratio and total chlorophyll and carotenoid concentrations of some indigenous Croatian cultivars and the international cultivars Chardonnay and Merlot are shown in Table 3. The leaf chlorophyll a/b ratio ranged from 1.6 to 6, with the highest values recorded after flowering, then decreasing during berry setting and remaining stable during bunch closure and veraison and then decreasing again during grape ripening. The highest leaf chlorophyll a/b ratio after flowering was recorded in the red cultivar Ljutun, while the lowest ratio was recorded in the white cultivar Debit. The highest leaf chlorophyll a/b ratio during berry setting was observed in the red cultivar Tribidrag, while the lowest ratio was observed in the white cultivar Maraština. Merlot showed the highest chlorophyll a/b ratio during bunch closure and veraison, the white cultivars Debit and Chardonnay during grape ripening, while the lowest ratio was observed in the red cultivar Ljutun.

The red cultivar Ljutun also had the lowest total chlorophyll and carotenoid concentration during grape development, apart from the total chlorophyll concentration during veraison and the total carotenoid concentration during bunch closure and grape ripening (Table 3). The cultivar Merlot showed the highest total chlorophyll and carotenoid concentration after flowering and during grape ripening, Maraština during berry setting and Graševina during veraison. The highest total chlorophyll concentration was observed during veraison, with an increase of 25% from flowering to veraison and a decrease of 52% during grape ripening, while the highest total carotenoid concentration was observed during berry setting, with an increase of 33% from flowering to berry setting and a decrease of 66% during grape ripening.

### 2.6. PCA

**Figure 5:**
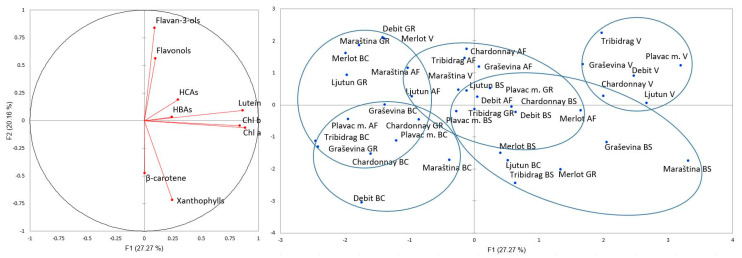
Principal component analysis (PCA) using the concentration of leaf carotenoids, chlorophylls and phenolic compounds (inserted vector diagram) of *Vitis vinifera* L. red cultivars Merlot, Plavac mali, Tribidrag and Ljutun and white cultivars Chardonnay, Maraština, Graševina and Debit. AF—after flowering, BS—berry setting, BC—bunch closure, V—veraison, GR—grape ripening.

The results of PCA applied to the eight cultivars at five phenological stages, based on the total concentration of leaf total flavan-3-ols, flavonols, HCAs and HBAs and chlorophyll a and b as well as the concentrations of β-carotene, lutein and xanthophylls, are shown in Figure 5. The first two principal components explain more than 47% of the total variability. With this limited explanation of variability (less than 50%), it must be assumed that other environmental conditions also contribute to the concentration of secondary metabolites in the leaf. The distance between the phenological stages bunch closure and veraison is most pronounced and is due to the higher concentrations of total HCAs, HBAs and the carotenoid lutein. One of the cultivars that shows great diversity is Merlot, which differs from the others in the phenological stages bunch closure, veraison and grape ripening. In addition, the effect of the cultivar is more pronounced than the effect of the phenological stage.

## 3. Discussion

Grape cultivars react to changes in the environment and cultivation methods and can change the composition of the leaves. This adaptability is called phenotypic plasticity [22]. To better understand plasticity in relation to leaf pigments and polyphenol synthesis, it is important to study grapevine cultivars under different environmental conditions and at different stages of development. Under conditions of increased sunlight, plants respond by adapting their metabolism at that stage of development to ensure normal vine growth and metabolism [23]. In other words, the adaptation is reflected in the fact that the typical developmental process is not affected, such as the physical parameters and the concentration of primary metabolites, but the perceived stress is managed with the synthesis of secondary metabolites that prevail at the specific developmental stage [24]. Secondary metabolites chlorophylls and carotenoids in grapevine leaves are upregulated in response to increased light exposure during the pre-veraison stage of berry development to protect photosynthetic membranes and ensure the maintenance of photosynthesis even under severe stress conditions [5]. This is called plasticity—the ability of a plant to respond and adapt to the environment and it is a function of both the cultivar and the environment. In other words, plants use solar radiation for photosynthesis and energy production, but also as a source of information about the environment [23]. Changes in microclimatic conditions can affect grapevine secondary metabolites through transcriptional changes that influence enzyme activity and biochemical reactions during grape ripening and mediate phenotypic plasticity at different developmental stages [24].

The composition of leaf metabolites varies considerably during the vegetative cycle, especially during the grape development stages due to environmental factors or the development of the plant (Figure 1, Figure 2, Figure 3 and Figure 4). The chlorophyll concentration in the leaves can vary greatly from cultivar to cultivar, with light exposure and temperature influencing the degradation processes differently for each cultivar [6]. The chlorophyll concentration in grapevine leaves in our study also varied depending on the phenological stage and cultivar (Figure 3, Table 3), with an increase from flowering to veraison and a decrease during grape ripening, which is consistent with the findings of other researchers [4,13]. Others suggested that chlorophyll accumulation increases rapidly with leaf expansion until flowering, then stabilizes and gradually decreases until the end of grape ripening [3,23]. Chlorophyll degradation is related to environmental stress, and the more rapid depletion of chlorophyll in leaves during vegetation may be related to higher temperatures and lower rainfall, as these types of events limit vegetative growth and accelerate leaf senescence [3,6,25]. Since the peak of photosynthetic activity is reached around flowering, most secondary metabolites are accumulated around this time and begin to degrade gradually from veraison to harvest [23]. In the study by Rafique et al. (2023) [6], the chlorophyll content index had the highest mean values at harvest, which is comparable to our results and was attributed to heavier rainfall just before maturity, which may have promoted chlorophyll synthesis.

The chlorophyll a/b ratio is a good indicator of leaf senescence, stress and damage to the photosynthetic apparatus [4]. The leaf chlorophyll a/b ratio ranged from 1.6 to 6, with an average value of 3.9 (Table 3), which is slightly higher than the values reported in other studies [3,4]. In general, plants with higher chlorophyll a/b ratios are better adapted to higher insolation, while lower values indicate stress conditions such as drought [4]. The highest values of chlorophyll a/b ratio were found after flowering, then decreased during berry setting and remained stable during veraison and then decreased again during grape ripening (Table 3), which is consistent with the results of other researchers [4].

The major carotenoids in grapevine leaves identified in this study were β-carotene, lutein and xanthophylls, which were abundant after flowering and then degraded during grape ripening (Figure 4). These findings are consistent with studies conducted so far [21]. In our study, the degradation of carotenoids started during bunch closure, while the degradation of chlorophylls started 3 weeks later, during veraison, which contrasts with the study of Filimon et al. (2016) [4] that found the opposite trend. The carotenoid trends observed here are consistent with reports on grape berries that carotenoids are highest early in the ripening period (before veraison) because light is necessary for their accumulation, whereas higher temperatures can increase carotenoid synthesis and carotenoid post-veraison degradation [26,27]. The highest carotenoid concentration was observed in most cultivars during berry setting and bunch closure (Figure 2), which could be related to the high sum of UV radiation observed during this time (Table 1), as increased UV radiation usually leads to increased levels of photoprotective carotenoids [19,20]. UV-B radiation has long been considered a general stressor for plants [23]. Instead, grapevine cultivars are relatively well adapted to UV exposure under field conditions, as ecologically relevant doses of UV-B radiation act as environmental modulators and regulate gene expression, metabolism and growth [28]. In grape berries, the xanthophylls zeaxanthin and lutein responded most strongly to UV-B radiation in the early stages of berry development. In the absence of UV-B, the berries required less zeaxanthin in exposed environments and less lutein epoxide in shaded environments [24]. Thus, the absence of UV-B could make grapes more susceptible to damage because they are less acclimatized than grapes exposed to UV-B radiation, which normally releases higher concentrations of photo-protective xanthophylls and flavonols [24]. Similar results were obtained in grape leaves, where xanthophyll cycle pigments (violaxanthin, antheraxanthin and zeaxanthin) increased with increasing UV radiation [14].

The total concentrations of flavan-3-ols and HCA in grapevine leaves were typically highest immediately after flowering and gradually decreased during grape ripening, while the total concentration of HBA in leaves of white cultivars was highest during bunch closure and in red cultivars during veraison (Figure 1 and Figure 2). This is consistent with the findings of Filimon et al. (2016) [4], who found that the concentration of polyphenolic compounds continues to decrease after veraison until leaf fall, probably due to degradation processes during leaf aging. In the research of Del-Castillo-Alonso et al. (2016) [13], leaf flavonols showed the lowest levels at pea size, increased at veraison and remained stable or increased further at harvest, while total HCA showed no significant changes over time. Other researchers [21,29] also reported that the polyphenol concentration in grapevine leaves varies depending on the cultivar and developmental stage of the leaf, with higher levels in immature leaves. Parameters such as environmental conditions (climate, soil) should also be considered to explain the variation in leaf polyphenol concentration. Polyphenols play an important role in the metabolism and physiology of grapevines and have the task of absorbing and shielding UV-B radiation [20].

When the plant is under stress, it produces free radicals, while at the same time synthesizing protective compounds such as polyphenols that protect it from free radicals [30]. A better way to cope with stress conditions is to synthesize more compounds that protect it from free radicals, and cultivars that have a great ability to synthesize more of these products are more likely to be more resistant to stress conditions such as drought, UV radiation or fungal attack [5,11,12,13]. One of the indigenous Croatian cultivars that showed higher levels of phenolic compounds is Debit, which had the highest levels of flavonols, HCA and HBA during grape ripening.

Excessive sunlight can damage grape leaves. Carotenoids accumulate in photosynthetic tissues as part of photosystem II to protect the tissue from light damage [7]. When too much oxygen is produced during photosynthesis in response to increased light intensity, it becomes toxic through the formation of free radicals; so, carotenoids, especially lutein and β-carotene, act as singlet oxygen scavengers [8]. They are also involved in dissipating excess light energy and converting it into heat. It is shown that the highest levels of carotenoid concentration were found in the international cultivar Merlot, but also in the indigenous cultivars Maraština and Graševina, especially during berry setting, bunch closure and veraison, indicating a major role of carotenoids in performing the protective function [25].

The chlorophyll a/b ratio and the concentration of various carotenoids, chlorophylls and phenolic compounds changed over the season with different temporal patterns (Table 3, Figure 1, Figure 2, Figure 3 and Figure 4). However, the relative amounts of each component can be altered differently, and different responses can occur depending on the stage of development [31] and cultivar [6]. Photosynthetic and photochemical parameters as diagnostic criteria for the identification of highly adaptive grape cultivars are widely used worldwide [25]. The different grapevine genotypic responses observed in this study provide an opportunity to select grapevine cultivars with physiological adaptations to synthesize secondary metabolites important for coping with stress conditions such as fungal attack, higher UV radiation and drought. In this study, we showed that the induction of the protective response is more pronounced in the cultivars Debit, Maraština and Graševina. Compared to Ljutun, Plavac mali and Tribidrag, these cultivars proved to be more adaptable under the conditions of the 2017 ripening period.

Due to the great diversity in physiological and biochemical characteristics between cultivars, as well as the high plasticity and adaptability under different growing conditions, it is worthwhile to study the responses of the grapevine that influence the plant’s performance under natural conditions. These results contribute to further the knowledge on grapevine plasticity and offer important advantages in terms of a better understanding of the dynamics of leaf pigments and phenolic compounds and the timing of their decline to define the behavior of Croatian indigenous red and white grapevine cultivars during grape development.

## 4. Materials and Methods

A one-year experiment (2017) was conducted with four red cultivars, including the international cultivar Merlot and three indigenous Croatian cultivars Plavac mali (VIVC No. 9549), Tribidrag (VIVC No. 9703) and Ljutun (VIVC No. 6878), and four white cultivars, including the international cultivar Chardonnay and three indigenous Croatian cultivars Maraština (VIVC No. 7262), Graševina (VIVC No. 13217) and Debit (VIVC No. 10423). The experiment was conducted at the experimental field Jazbina (University of Zagreb, Faculty of Agriculture, 45°51′ N, 16°0′ E), which is characterized by a moderately warm and rainy continental climate. All indigenous Croatian cultivars were planted in the national vine collection at the site and the vines were planted from 2001 to 2005 at a spacing of 1.1 × 2.1 m (4329 vines/ha) and unilateral Royat cordon trained, leaving 10 buds per vine. The Merlot vines were planted in 2005, while the Chardonnay vines were planted in 1995, both at 1.2 × 2.1 m spacing (3968 vines/ha) and double Guyot trained, leaving 24 buds per vine. The rows were east–west oriented. The fruit-bearing wire was placed at a height of 80 cm above the ground and two additional sets of interception wires were placed at 40 cm from the bearing wire. The maximum canopy height was 180 cm. All experimental vines were grafted onto the rootstock ‘SO4’. The soil was an anthropogenic pseudogley with a clay texture. Viticultural practices were conducted as usual for this wine-growing region. Shoots that exceeded the height of the trellis were cut back to 20 cm above the last wire 4 weeks before veraison. The vines were not irrigated.

The data for daily air temperatures and precipitation were obtained from a weather station in the experimental field (Pinova Meteo, Pinova d.o.o., Čakovec, Croatia). UV-A + UV-B radiation within the foliage at cluster level was continuously monitored throughout the growing season (frequency of 1 s) and measured using the Kipp & Zonen SUV5 UV radiometer connected to the Campbell Scientific CR-3000 3 G data logger with solar charging. The mean values of air temperature and precipitation were based on the average 60-min values, while UV-A + UV-B radiation was calculated as the sum of the average 60-min values calculated from the data collected during the season.

The phenological stages were monitored weekly and the phenological stages—full bloom, bunch closure, veraison and mid-ripening—were determined for each cultivar. To determine a respective phenological stage, it must be recorded on at least 50% of the vines. The phenological stage of full bloom was recorded when 50% of caps fell (E-L stage 23) [32], bunch closure when the berries began to touch (E-L stage 32), veraison when 50% of the berries changed color (E-L stage 32) and mid-ripening when the grapes had intermediate Brix values (E-L stage 36). The leaf samples were taken during the grape development period, which began immediately after flowering, continued every 3 weeks and ended with the final stage of grape ripening, as not all the cultivars reached grape maturity due to their different ripening dynamics. The sampling dates were as follows: 14 June, 4 July, 21 July, 14 August and 5 September, which coincided with the phenological stages—end of flowering, during berry set, bunch closure, veraison and the last stage of grape ripening. At the time of the specific leaf sampling date, all cultivars reached the respective phenological stage.

The average leaf sample consisted of three to five leaves from different vines, taken from the position opposite the first cluster on the shoot. After sampling, the leaves were packed in paper bags and stored at −20 °C until further analysis. The leaf samples were then freeze-dried without the stalk removed (Alpha 1–2 LDPlus Martin Christ, Osterode am Harz, Germany). After freeze-drying, the leaves were ground to a fine powder, which was then sieved to remove hairs.

For the analysis of carotenoids and chlorophylls in the leaf samples, samples weighing 20 mg ± 1 mg were weighed and 1 mL of solvent consisting of acetone and 0.1% BHT (butylated hydroxytoluene) was added. The resulting extraction mixture was stirred for 45 min. At the end of stirring, centrifugation at −4 °C and 28,900× *g* was performed. The resulting supernatant was separated from the precipitate and evaporated to dryness in a nitrogen stream. Prior to analysis by high-performance liquid chromatography (HPLC), the precipitate was dissolved in 200 μL of a solvent consisting of ethyl acetate and methanol (1:4, *v*/*v*) with 0.1% (*w*/*v*) BHT. The resulting extract was analyzed using a high-performance liquid chromatograph (Agilent 1100 series). A YMC carotenoid column (4.6 × 250 mm; 5 µm particle size (YMC, Kyoto, Japan)) with gradient elution using a mixture of methanol, water, ammonium acetate and triethylamine (solvent A) was used for the separation of each analyte, while B was a mixture of methyl tert-butyl ether and triethylamine at a flow rate of 1 mL/min. A sample volume of 20 μL was injected during the analysis, and a column temperature of 25 °C was used. Chlorophyll a was detected at 420 nm, lutein and β-carotene at 450 nm and chlorophyll b at 470 nm. All analyses were performed in triplicate, and the result was expressed in mg/g of dry leaf weight (DW).

The method for the determination of polyphenolic compounds in grapevine leaves was described in [10]. In brief, leaf samples were placed in glass vials. A total of 10 mL of extraction solvent was added to the weighed samples weighing 180 ± 1 mg. It consisted of 20% acetonitrile, 1% formic acid and 79% water. The prepared extraction mixture was stirred for 90 min at a temperature of 48 °C on a magnetic stirrer. The resulting supernatant was filtered through a membrane filter (PTFE (Teflon), 0.45 µm) and then subjected to HPLC analysis. An Agilent 1100 series high-performance liquid chromatograph was used for sample analysis. The device consists of an automatic autosampler, a detector with a series of diodes, a binary pump and a fluorescence detector, bottles for the mobile phase and a computer to control the HPLC program. A Luna phenyl-hexyl column (4.6 × 250 mm; 5 μm particle size (Phenomenex, Torrance, CA, USA)) was used to separate the individual phenolic compounds. In this analysis, a 0.5% (*v*/*v*) aqueous solution of phosphoric acid was used as the first solvent and a solution of acetonitrile, water and phosphoric acid (50:49.5:0.5; *v*/*v*/*v*) as the second solvent. The flow rate was 0.9 mL/min, the volume of the sample injected was 20 μL and the column temperature was 50 °C. The analyses were performed as three replicates, and the results are expressed in mg/g of dry leaf weight.

All data were analyzed by one-way analysis of variance and the LSD test using XLSTAT software Version.2020.3.1. (Addinsoft, New York, NY, USA). Principal component analysis (PCA) was applied to the data set to distinguish individual carotenoids and chlorophylls as well as groups of phenolic compounds based on cultivar and the phenological stage. All data for PCA were previously transformed to standardize the data for each cultivar (*n* − 1).

## Figures and Tables

**Table 1:** Daily mean air temperature, sum of UV-A + UV-B radiation and precipitation in 2017, Jazbina experimental station.

Month	Air Temperature (°C)	UV Radiation (W/m^2^)	Precipitation (mm)
May	17.77	890.69	91.0
June	22.39	1396.36	108.0
July	23.87	1610.66	76.4
August	24.21	1673.75	48.1
September	15.34	777.36	221.7

**Table 2:** Start dates and days after flowering of key phenological stages in different grapevine cultivars.

Cultivar	Full Bloom	Bunch Closure	Veraison	Mid-Ripening
Chardonnay	30 May	13 July (44 DAF)	7 August (69 DAF)	27 August (89 DAF)
Graševina	31 May	13 July (43 DAF)	9 August (70 DAF)	29 August (90 DAF)
Tribidrag	6 June	15 July (39 DAF)	8 August (63 DAF)	29 August (84 DAF)
Merlot	1 June	17 July (46 DAF)	8 August (68 DAF)	2 September (93 DAF)
Maraština	5 June	20 July (45 DAF)	14 August (70 DAF)	1 September (88 DAF)
Debit	6 June	21 July (46 DAF)	14 August (69 DAF)	4 September (90 DAF)
Ljutun	1 June	21 July (50 DAF)	14 August (74 DAF)	5 September (96 DAF)
Plavac mali	1 June	20 July (49 DAF)	14 August (74 DAF)	5 September (96 DAF)

DAF—days after flowering.

**Table 3:** Changes in leaf chlorophyll a/b ratio and total chlorophyll and total carotenoid concentration during grape development stages of *Vitis vinifera* L. red cultivars Merlot, Plavac mali, Tribidrag and Ljutun and white cultivars Chardonnay, Maraština, Graševina and Debit.

Phenological Stage	Cultivar	Chl a/b	Total Chl	Total Car
After flowering	Merlot	5.5 c	2.18 a	1.37 a
	Plavac m.	5.4 c	1.03 e	0.61 e
	Tribidrag	3.9 de	1.80 b	0.74 d
	Ljutun	6.0 a	0.99 e	0.56 e
	Chardonnay	4.4 d	1.67 c	0.96 b
	Maraština	5.7 b	1.78 b	0.96 b
	Graševina	3.8 e	1.82 b	0.97 b
	Debit	1.6 f	1.17 d	0.83 c
Berry setting	Merlot	3.9 bc	1.87 d	1.38 b
	Plavac m.	3.6 bc	1.31 f	0.75 d
	Tribidrag	4.9 a	2.11 c	1.18 c
	Ljutun	4.6 b	0.44 g	0.53 e
	Chardonnay	3.6 bc	1.64 e	1.16 c
	Maraština	3.4 c	3.29 a	1.75 a
	Graševina	4.0 bc	2.47 b	1.41 b
	Debit	4.2 bc	1.65 e	1.11 c
Bunch closure	Merlot	5.2 a	1.09 d	0.79 c
	Plavac m.	4.8 b	1.21 c	0.79 c
	Tribidrag	2.9 cd	1.29 b	0.64 d
	Ljutun	2.4 d	1.02 e	0.73 cd
	Chardonnay	3.2 cd	1.35 b	0.96 b
	Maraština	2.7 d	2.09 a	0.82 c
	Graševina	4.8 b	1.31 b	1.07 a
	Debit	4.0 bc	1.16 c	0.75 c
Veraison	Merlot	5.4 a	1.24 f	0.86 b
	Plavac m.	3.7 c	2.18 c	0.80 c
	Tribidrag	4.0 c	2.02 d	0.83 bc
	Ljutun	1.9 e	1.54 e	0.57 e
	Chardonnay	2.9 d	2.09 cd	0.68 d
	Maraština	4.8 b	2.11 cd	1.11 a
	Graševina	5.2 ab	2.80 a	1.11 a
	Debit	2.5 de	2.54 b	0.55 e
Grape ripening	Merlot	3.6 b	2.37 a	1.16 a
	Plavac m.	3.4 bc	1.44 c	0.85 b
	Tribidrag	2.8 de	1.53 b	0.84 b
	Ljutun	2.4 e	0.36 f	0.47 e
	Chardonnay	4.5 a	1.57 b	0.61 d
	Maraština	3.5 bc	1.26 d	0.56 d
	Graševina	3.1 cd	1.30 d	0.74 c
	Debit	4.1 a	1.03 e	0.35 f

Chl = chlorophyll, Car = carotenoids; total chlorophylls and total carotenoids are expressed in mg/g DW; letters indicate statistically significant difference between variables within sampling dates, with LSD test (*p* < 0.05).

## Data Availability

Data are contained within the article.
